# Knockdown of *armc3* Impairs Motile Cilia Function in *Schmidtea mediterranea*

**DOI:** 10.17912/micropub.biology.001887

**Published:** 2026-01-16

**Authors:** Chayanika Gogoi, Rachel Pitt, Kate Mazur, Ramyasri Naraharisetti, Kristen Johnson

**Affiliations:** 1 Department of Life Sciences, University of New Hampshire at Manchester, Manchester, New Hampshire, United States

## Abstract

Cilia are microtubule-based structures lining epithelial surfaces of many organs and play an essential role in diverse metabolic and developmental processes. Structural or functional disruptions of cilia can lead to ciliopathies affecting multiple organs. Knocking down *armc3* in *Schmidtea mediterranea* revealed reduction in cilia length of 48.9% compared to the control, accompanied by 63.7% reduction in gliding speed. Additionally, knockdown planaria displayed abnormal cilia distribution, particularly in the anterior region. These findings suggest that ARMC3 is essential for maintaining proper motile cilia structure and function and highlight its potential relevance for understanding ciliopathies in humans.

**Figure 1. Cilia formation and swim speed were significantly reduced in planaria with knockdown of the armc3 gene:**
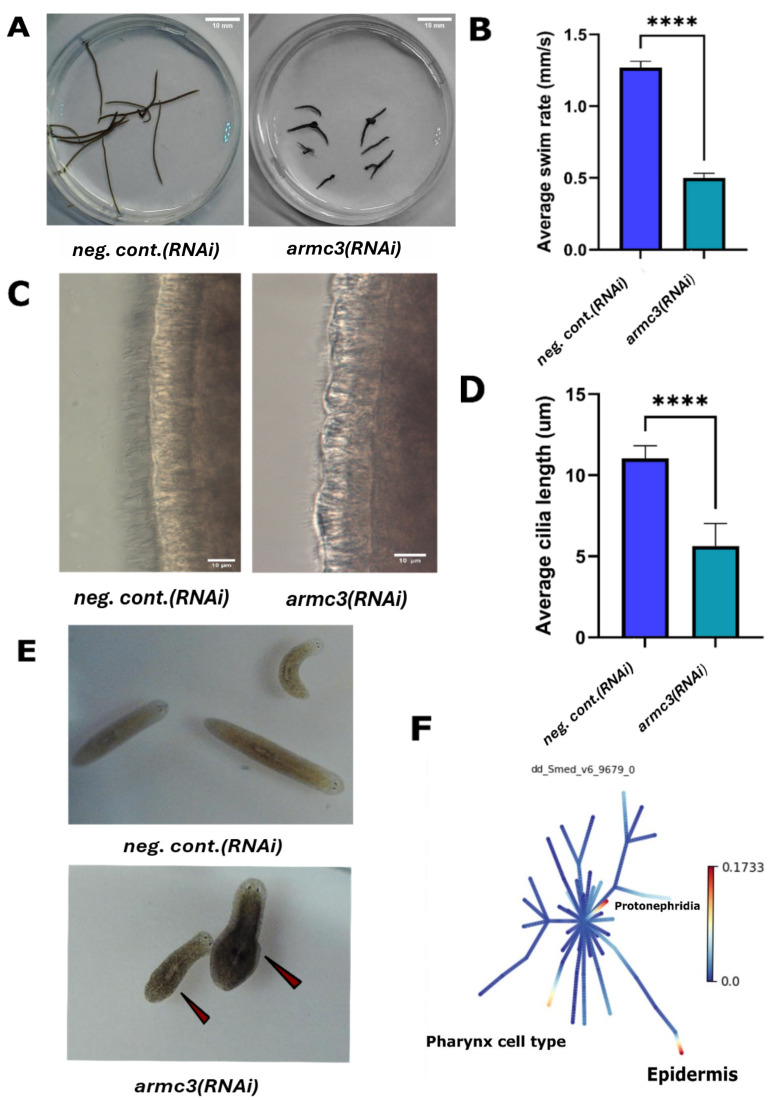
**
(A) Pathway tracing of *armc3(RNAi)* worms showing reduced distance traveled over 20 seconds in Z-projection images** . Overlays of sequential frames from 20 second video segments, illustrating the distance travelled by individual flatworms in *neg. cont.(RNAi)* and *armc3(RNAi)* worms. Z Projection within ImageJ software was used to perform the path tracing analysis. **(B) RNAi-mediated knockdown of *armc3* significantly impairs planarian gliding.** *Neg. cont.(RNAi)* worms exhibited a normal swim speed (1.30 mm/s), whereas *armc3(RNAi)* worms showed a 63.67% decrease in swim speed (0.47 mm/s). n= 27. Error bars represent the standard error of the mean (SEM). ***p < 0.001. ( **C)** **DIC Microscopy Images Showing Reduced Cilia Length After Gene Knockdown** . Differential interference contrast (DIC) microscopy images of cilia show a visible reduction in length following knockdown of the *armc3* gene compared to *neg. cont.(RNAi)* . **(D) RNAi-mediated knockdown of *armc3* in planarians results in reduced cilia length.** Quantitative analysis revealed a 48.87% decrease in cilia length in *armc3(RNAi)* worms (5.64 µm) compared to *neg. cont.(RNAi)* (11.04 µm). n=10. Error bars represent SEM. ****p < 0. 0001. **(E) Bloated phenotypes in *armc3* knockdown worms.** Image on the top represents *neg. cont.(RNAi)* and on the bottom *armc3(RNAi)* worms which show bloating. Red arrows indicated bloated areas in *armc3(RNAi)* worms. **&nbsp;(F) Single-Cell RNA-Seq Expression of *armc3* in Planarian Cell Clusters:** Single-cell RNA-seq shows high expression of dd_smed_v6_9679_0 ( *armc3* ) in clusters of Protonephridia and pharynx cell with moderate expression of epidermal progenitors, where ciliated cells originate (Plass et al., 2018).

## Description

Armadillo Repeat Containing 3 ( *ARMC3* ), encodes a protein containing Armadillo/beta-catenin-like repeat domains (NIH, 2024). *ARMC3* was identified as one of six poorly characterized genes within a 248-gene ‘motile cilia’ signature, derived from Forkhead box protein J1 (FOXJ1) co-expression analysis across multiple human tissues including lung, brain, fallopian tube, endocervix, and testis in the Genotype-Tissue Expression (GTEx) project (Patir et al., 2020). This 2020 study confirmed *ARMC3* expression in the motile-ciliated ependymal cells lining the brain ventricles using&nbsp; *in-situ* &nbsp;hybridization data from the Allen mouse brain atlas and single cell RNASeq (Patir et al., 2020).&nbsp;

Cilia are hair-like protrusions that arise from the surface of many eukaryotic cells. Structurally, cilia are composed of microtubules that undergo continuous polymerization and depolymerization. The core structure of cilia is evolutionarily conserved and is comprised of three main components: the basal body, the axoneme, and the ciliary membrane (Hoyer-Fender, 2013). Three main types of cilia exist: primary, nodal, and motile that carry out a diverse array of physiological functions in the cell, including signal transduction, sensing, and motility, respectively. Additionally, flagella (present in sperm) contain similar structures as motile cilia. Based on the architecture of their axoneme, motile cilia feature a characteristic ‘9+2’ microtubule arrangement, where nine outer doublets surround a central pair of singlet microtubules (Klena & Pigino, 2022). Structural elements such as dynein arms, nexin links, and radial spokes drive ciliary beating and provide mechanical support, allowing motile cilia to generate directional fluid flow and facilitate fluid movement such as in the respiratory tract and reproductive tract (Breslow & Holland, 2019). Ciliopathies, which arise from genetic mutations affecting ciliary structure or function, lead to diseases involving the eye, kidney, brain, skeleton, and reproductive organs and can result in chronic respiratory problems, hydrocephalus, and infertility (Reiter & Leroux, 2017; Higgins et al., 2019).

Prior studies have made functional connections between *ARMC3* and sperm motility.&nbsp; For example, ARMC3 was identified as a proteomic marker of high fertility in bovine specimens and was associated with highly motile sperm (D’Amours et al., 2019). Deletion of *ARMC3* exon 11 in cattle leads to premature termination of translation and causes sperm defects and male infertility (Pausch et al., 2016).&nbsp; In humans, ARMC3 dysfunction has been associated with asthenozoospermia, a male infertility condition caused by reduced sperm motility due to exon skipping in the *ARMC3* transcript (Rahim et al., 2024). ARMC3 has been associated with axonemal regulators such as SPAG6 (which also contains armadillo repeats), known to cause sperm flagellar malformations and hydrocephalus (due to nodal cilia defects) in mice (Lonergan et al., 2006; Sapiro et al., 2002). In addition, ARMC3 has been identified as the mammalian homolog of yeast Vac8 with conserved roles in autophagy initiation. Deletion of *ARMC3* in mice results in male infertility by disrupting ribophagy, reducing mitochondrial energy, which results in immotile sperm flagella, underscoring a germline-specific autophagic role (Lei et al., 2021).

However, despite these insights, the direct functional role of ARMC3 in motile cilia remains untested especially in the context of multiciliated cells. Current evidence is largely derived from transcriptomic associations, evolutionary comparisons, or sperm-specific studies, leaving unclear whether ARMC3 contributes to motile cilia structure or function. Our study addresses this gap by examining the effects of *ARMC3* knockdown on motile cilia in the context of multiciliated cells to determine its functional role in broader ciliary biology.

In this instance, *Schmidtea mediterranea* (freshwater planaria) serves as an ideal model organism. The planarian *S. mediterranea* is a low-cost, easily maintained laboratory model and is one of few multicellular organisms with an external multiciliated epithelium. Its ventral epithelial cells bear many cilia that beat against a secreted mucus layer, in coordination with muscle contraction, to drive locomotion—similar to mucus transport by cilia in the human airway. Widely studied for its regenerative abilities, *S. mediterranea* now has a sequenced genome and robust molecular tools, including RNAi for gene function analysis. Regenerative capacity and the ease of knocking down the genes through RNA interference (RNAi) has made it an ideal organism for studying cilia gene related knockdown. (Rompolas et al., 2010; Rabiasz & Ziętkiewicz, 2023). Additionally, protonephridia, excretory and osmoregulatory structures, function by using the movement of cilia to filter waste products and excess water from the body fluid.&nbsp; Disruption in the cilia in this organ can result in fluid retention and planaria bloating (Thi-Kim Vu et al., 2015). In this study, we used RNA interference to knock down the planarian homolog of *ARMC3* (dd_Smed_v6_9679_0_1) to investigate its role in ciliary architecture and function.

RNAi-mediated knockdown of the planaria homolog of human *ARMC3* in *S. mediterranea* produced clear phenotypic changes related to ciliary function and morphology. One-week post-RNAi treatment, worms were assayed using microscopy techniques for cilia length and motility rate.&nbsp; Twenty-second video segments were processed using Fiji to generate Z-projection images which demonstrated reduced travel distances in *armc3(RNAi)* worms compared to neg. cont.(RNAi) worms confirming impaired locomotion ( Fig. 1A, Extended data video 1- neg.cont.(RNAi) 20sec, Extended data video 2- armc3(RNAi) 20sec). Quantitative analysis of gliding behavior revealed a 63.67% decrease in swimming velocity, from 1.30 mm/sec in neg. cont.(RNAi) worms to 0.47 mm/sec in *armc3(RNAi)* ( Fig. 1B ).&nbsp; The worms also exhibited an inch worming phenotype, relying on muscular contractions for movement instead of smooth gliding driven by ventral cilia (Extended data video 3-neg.cont.(RNAi), Extended data video 4- inch worming phenotype of armc3(RNAi) worms).

Differential Interference Microscopic (DIC) analysis revealed substantial reductions in cilia length in *armc3(RNAi)* worms ( Fig. 1C ). Quantification of cilia length confirmed a decrease from 11.04 µm in neg. cont.(RNAi) to 5.64 µm in the *armc3(RNAi)* group, representing a 48.87% reduction ( Fig. 1D ). Additionally, qualitative observations of uneven cilia growth were noted. In addition to structural and motility defects, *armc3(RNAi)* worms exhibited bloating in the abdominal region, a phenotype indicative of defective protonephridia, the planarian excretory system. This swelling suggests compromised fluid regulation, likely due to cilia dysfunction in the flame cells in the protonephridia ( Fig. 1E ).

Supporting these findings, planaria single-cell transcriptome analysis shows high *armc3* expression in planarian protonephridia, pharyngeal cell clusters, and in epidermal progenitors, the origin of ciliated epidermal cells ( Fig. 1F ) (Plass et al., 2018). This expression profile correlates with the phenotypes observed upon gene knockdown, including reduced motility, shortened cilia, and swollen abdomen region. Our findings in planaria provide functional evidence of the role of ARMC3 in cilia assembly and performance, strengthening its relevance to human ciliopathies.

Our study demonstrates that ARMC3 is essential for maintaining normal cilia length and function in multiciliated cells in planaria. RNAi knockdown of *armc3* results in impaired locomotion, shortened and sparse cilia, and fluid imbalance, mirroring cilia-related disorders seen in humans. These results highlight the utility of *S. mediterranea* as a model for studying conserved ciliary genes and suggest that ARMC3 plays a critical and conserved role in motile cilia biology. Understanding ARMC3 function may aid in elucidating the molecular mechanisms underlying human ciliopathies and reproductive disorders. &nbsp;Together, these findings identify a motile cilium related function for ARMC3 and confirm its place within a conserved motile cilia program across tissues in humans (Patir et al., 2020).

## Methods

**Planarian Maintenance and Feeding Protocol** Planarians were maintained in a dark environment in vessels containing planarian water at room temperature made as follows: 1.6 mM NaCl, 1.0 mM CaCl _2_ , 1.0 mM MgSO _4_ , 0.1 mM MgCl _2_ , 0.1 mM KCl, 1.2 mM NaHCO _3_ . Worms were fed once weekly with organic beef liver paste. After two hours of feeding, remaining food was removed using plastic transfer pipettes, and a small volume of fresh planarian water was added to top off the vessel. A complete water change was performed every two days to remove accumulated excretory waste and maintain optimal water quality (Rompolas et al., 2009).

**RNAi Plasmid Cloning:** The ARMC3 FASTA gene transcript (dd_smed_v6_9679_0_1) for *Schmidtea mediterranea* was obtained from the PlanMine database (Rozanski et al., 2019). Primer design was performed using Benchling [Biology Software] (2025) to generate a gene fragment, with additional homologous ends incorporated for insertion into the pPR-T4P plasmid vector. Genes of interest were amplified from cDNA and cloned through ligation-independent cloning. For ARMC3, a 747 bp fragment was amplified using the following primers: ARMC3-fwd, CATTACCATCCCGggccttgggatatctttgcc; ARMC3-rev, CCAATTCTACCCGattccaatagcctgtgcagc. (Sequences for homology arms are indicated in capital letters and gene specific primer sequences in lowercase.)

**Insert preparation:** Planaria were homogenized using sonication and RNA was collected (Zymo Direct-Zol RNA Purification Kits). cDNA was prepared (Invitrogen SuperScript III First-Strand Synthesis System). Gene specific PCR was then carried out using either Q5 polymerase or Taq Polymerase (New England Biolabs) and successful amplification of fragments was verified using gel electrophoresis. The PCR product was purified (Zymo Clean and Concentrate kit), and its concentration was measured (Denovix) to calculate insert and plasmid backbone ratios (2:1) for cloning. Inserts were cloned into pPR-T4P plasmid backbones using InFusion assembly (InFusion Snap Assembly EcoDry Master Mix) according to manufacturer’s instructions. Cloned pPR-T4P constructs were transformed into *E. coli DH5α* cells by heat shock and plated on LB-kanamycin plates. Colony formation confirmed plasmid uptake, and selected colonies were screened by PCR with M13 primers and gel electrophoresis to verify gene-specific insert incorporation. A positive colony for each gene was cultured overnight in LB-kanamycin, and plasmid DNA was purified (ZymoPure Mini Kit). Successfully assembled plasmids were then nanopore sequenced (Plasmidsaurus) prior to RNAi food preparation (Adler & Alvarado, 2018).

**RNAi Feeding:** RNAi plasmids (pPR-T4P + gene fragment) were transformed into *E. coli* HT115, an RNAse III-deficient strain enabling dsRNA production (Timmons et al., 2001). Overnight cultures were diluted 1:50 into fresh LB-KAN broth and incubated ~5 hours to OD₆₀₀ 0.5–0.7. Expression was induced with 1 mM IPTG for 2 hours with shaking. Cultures were centrifuged, supernatant removed, and pellets resuspended in a 2:1 liver/planarian water mixture at 1:300 of the initial culture volume. The mixture was aliquoted (~60 μL per tube) and frozen at –80°C. Following a one-week starvation, three experimental plates (10 planaria/plate per RNAi gene) were fed six times over two weeks. The *C.elegans* gene UNC-22 with no known homolog in the planarian genome was used as a negative control [neg. cont. (RNAi)].

**After 7 days of the last feeding, assays were undertaken as follows:**

**Cilia Phenotype Analysis** : Planarians were immobilized by incubation in a relaxant solution (1% HNO _3_ , 0.8325% formaldehyde, and 50 mM MgSO _4_ ) for 5 minutes prior to mounting on prepared slides. Specimens were imaged using differential interference contrast (DIC) optics on a Zeiss LSM 510 Meta confocal microscope. For each condition, five worms were analyzed. Images were captured from both the left and right sides of the head region, near the eyes, where cilia are more densely distributed. From each image, the lengths of 10 individual cilia were measured, resulting in 10 data points per image. Measurements were performed using Fiji software (Schindelin et al., 2012) to calculate the average cilia length for both control and RNAi-treated planarians.

**Gliding Assays** : Planarian locomotion was recorded over a 20-second period using an Andonstar AD246S-M HDMI Digital Microscope. Videos were analyzed in Fiji, where z-projection was applied to generate tracks of individual worms. The lengths of these tracks were measured to calculate average swimming speed (mm/s).

**Statistical Analysis:** Gliding velocity and cilia length were analyzed using Fiji software. Graphpad Prism Software version 10.0 was used to analyze the data, employing Welch’s t-test to analyze data variance with significance defined as p<0.05.

## Reagents

**Table:** 

**Category**	**Reagents**	**Use/Notes**	**Source of Reagent**
**Plasmids & Vectors**	pPRt4p plasmid	Expression vector backbone for cloning & RNAi experiments	Gifted by Jason Pellettieri
**Bacterial Strains**	HT115 *E. coli*	RNAi feeding strain (RNase III deficient, used in dsRNA expression)	Gifted by Jason Pellettieri
	NEB 5-alpha *E. coli*	High efficiency cloning strain for plasmid propagation	New England Biolabs
**Primers**	ARMC3-fwd	CATTACCATCCCGggccttgggatatctttgcc	Invitrogen
	ARMC3-rev	CCAATTCTACCCGattccaatagcctgtgcagc	Invitrogen

## Data Availability

Description: Extended Data Video 1. Resource Type: Audiovisual. DOI: https://doi.org/10.22002/br21k-ksh75 Description: Extended Data Video 2. Resource Type: Audiovisual. DOI: https://doi.org/10.22002/bgdp2-gxe39 Description: Extended Data Video 3. Resource Type: Audiovisual. DOI: https://doi.org/10.22002/gjsvq-9g151 Description: Extended Data Video 4. Resource Type: Audiovisual. DOI: https://doi.org/10.22002/rm3pk-gny98
